# Feature-Based Information Retrieval of Multimodal Biosignals with a Self-Similarity Matrix: Focus on Automatic Segmentation

**DOI:** 10.3390/bios12121182

**Published:** 2022-12-19

**Authors:** João Rodrigues, Hui Liu, Duarte Folgado, David Belo, Tanja Schultz, Hugo Gamboa

**Affiliations:** 1 Laboratory for Instrumentation, Biomedical Engineering and Radiation Physics, NOVA School of Science and Technology, Campus da Caparica, 2829-516 Caparica, Portugal; 2 Cognitive Systems Lab, University of Bremen, Bibliothekstraße 1, 28359 Bremen, Germany; 3 Associação Fraunhofer Portugal Research, Rua Alfredo Allen 455/461, 4200-135, Porto, Portugal

**Keywords:** automatic segmentation, unsupervised segmentation, novelty function, human activity recognition, biosignal processing, self-similarity matrix, clustering, information retrieval, data mining

## Abstract

Biosignal-based technology has been increasingly available in our daily life, being a critical information source. Wearable biosensors have been widely applied in, among others, biometrics, sports, health care, rehabilitation assistance, and edutainment. Continuous data collection from biodevices provides a valuable volume of information, which needs to be curated and prepared before serving machine learning applications. One of the universal preparation steps is data segmentation and labelling/annotation. This work proposes a practical and manageable way to automatically segment and label single-channel or multimodal biosignal data using a self-similarity matrix (SSM) computed with signals’ feature-based representation. Applied to public biosignal datasets and a benchmark for change point detection, the proposed approach delivered lucid visual support in interpreting the biosignals with the SSM while performing accurate automatic segmentation of biosignals with the help of the novelty function and associating the segments grounded on their similarity measures with the similarity profiles. The proposed method performed superior to other algorithms in most cases of a series of automatic biosignal segmentation tasks; of equal appeal is that it provides an intuitive visualization for information retrieval of multimodal biosignals.

## 1. Introduction

In recent years, the continuous increase in accessible wearable technology has contributed to a significant amount of data available. Continuous data collection from wearable devices through mobile phones, smartwatches, hearables, wristbands and other non-invasive wearable sensors has provided a valuable volume of information. As reported in Tankovska et al., wearable devices’ usage has more than doubled between 2016 and 2019, reaching 722 million, each of which relies on high-quality data acquisition and processing [1].

The data recorded by wearable devices carry information in the form of time series, which typically has an ordered structure. The displayed mechanics may be the expected or consequence of the nature of the acquisition environment, e.g., controlled laboratory experiments or natural scenarios. Researchers, such as data scientists, are interested in understanding the structure of the recorded signals, the meaning behind them, and the influences of the context. For instance, in the context of audio signals from musical pieces, it is helpful to acknowledge the different structural components for distinguishing the intro from the chorus and the bridge. Shifting to biosignals-related domains, such as Human Activity Recognition (HAR), the *melody* can change and evolve from walking to jogging (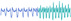)—this Accelerometer (ACC) signal contains two main periodic regimes, which could be segmented as WW…W and JJ…J. Another example comes from the Electrocardiogram (ECG), a typical physiological signal. The standard cyclic nature of the ECG, interesting to most users, may be affected by several sources, such as motion artefacts, muscular contractions or even symptomatic events. For instance, the signal piece 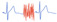 has two cycles of an ECG disturbed by noise for which we can interpret that it has three segments, and the first segment is very similar to the third one, i.e., *ABA*.

The examples mentioned above manifest the relevance and importance of the following approaches:

- Novelty segmentation: to identify significant changes in the signal’s behaviour.
- Periodic segmentation: to detect the presence of repeating cyclic patterns.
- Labelling: to measure how similar the segments are between each other.

This study explored and proposed the solution to these three problems mentioned above, inspired by a method used for audio signal analysis and thumbnail generation [2,3,4,5]. A moderately similar operation was introduced for speech recognition [6]. Surprisingly, such a method has not yet been extended to other types of time series domains that could greatly benefit from it [7]. The method uses a feature-based Self-Similarity Matrix (SSM) of (multidimensional) time series, from which visual and analytical information is rendered to perform the segmentation process and associate subsequences of the time series with each other.

**Figure 1:**
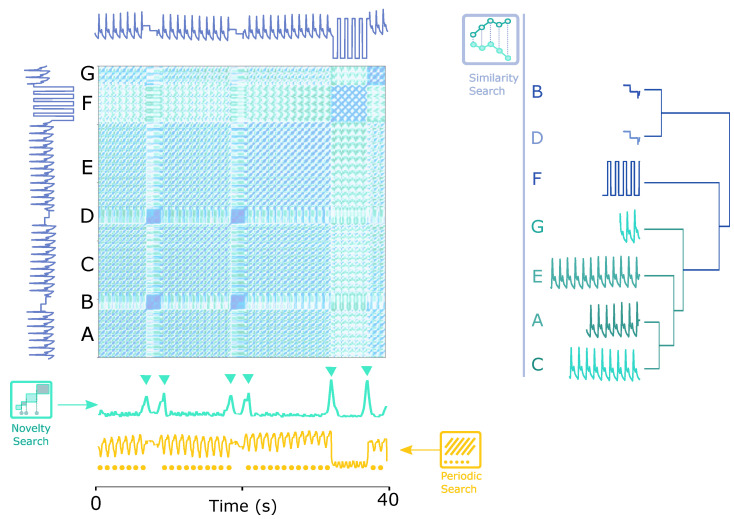
A visual structural description of functions on a time series for retrieving relevant events, segmenting, and associating the previously segmented subsequences based on the feature-based SSM. (**Left**): an Arterial Blood Pressure (ABP) signal’s SSM representing the pairwise similarity between subsequences, where the “novelty search” signal in green below the matrix demonstrates the novelty function and the “periodic search” signal in orange depicts the similarity function; (**right**): the clustering procedure of the novelty function-based segmented subsequences according to their similarity values in the SSM.

Figure 1 shows a teaser example of how the SSM works. The time series has seven segments (A, B, C, D, E, F and G), divided into three different groups of sub-sequences (1: [A, C, E, G], 2: [B, D] and 3: [F]), among which groups 1 and 3 are periodic.

As conceptualized in Figure 1, by applying the proposed solution, the following tasks were handled intuitively:

- **Novelty search**: the signal is segmented into homogeneous groups by the novelty function.
- Periodic search: the periods in the signal are segmented by the similarity function.
- Similarity measurement: the similarity between segments can be reasonably explained by the colour of the corresponding sub-sequence pair on the matrix, and the values can be used to cluster the segments by similarity, as illustrated on the dendrogram of Figure 1 (right).

In this article, the effectiveness of the proposed method as a powerful tool for unsupervised signal analysis will be explored in a set of examples in different domains and levels of information (*novelty*, *periodicity* and *similarity*). The results of the novelty segmentation will be validated and compared to public benchmarks.

### 1.1. Essentials

The segmentation of time series has a subset of extensive and relevant applications, transverse to all domains. The task of dividing a time series into segments is context- and purpose-oriented, depending on different interest levels of instants or events for correctly selecting breaking points on the time series. Before delving into more details, the main concepts frequently used in this study must be clarified:

**Time Series:** A time series is a sequence of real values ordered in time with length n∈N: $T=(t_{1},t_{2},\ldots ,t_{n})$. A biosignal is a category of time series. Several data domains rely on the multidimensional time series acquisition from one sensor’s multiple axes, such as an ACC’s three directions, or multiple sources, such as an Inertial Motion Unit (IMU) that fuses three different sensors.
**Multidimensional Time Series:** A multidimensional time series is a set of k∈N time series belonging to the same acquisition: $\left\{T_{1},T_{2},\ldots ,T_{k}\right\}$. Segments of interest, called subsequences, are often searched inside a time series.
**Subsequence:** A subsequence is a segment of a (*multidimensional*) time series with size w∈N, starting from a given position *i* and ending at position *i + w*. Therefore, two instants defined as events, delimit a subsequence in time.
**Event:** An event is an instant in time *e* that indicates the presence of a relevant occurrence in the time series. Multiple events segment the time series into several subsequences of different lengths. Hence, event detection is often considered time series segmentation or change point detection [8]. To be clear, we will use the terms event detection and segmentation when discussing our methods, but we can eventually use the term change point detection when comparing with other methods.
**Moving Window:** A moving window is a process of sliding along a time series *T* to apply a specific method on each subsequence it hovers, a common strategy used in time series data mining to find relevant subsequences and events. The window has, similar to the subsequence, a predefined size w∈N, which starts at a given position *i* and ends at position *i* + *w*. The process of moving windows is iterative, and windows can overlap each other. The following window will start at *i* + *o*, where *o* ∈[1,w] is the overlapping size (o=1 for a total overlap and o=w for no overlap). On each moving window from each subsequence of the (multidimensional) time series, features can be extracted to form a feature series.
**Feature Series:** A feature series is a feature representation of a time series with size $m=\frac{n}{w-o}$ that depends on the overlap size o∈N of the moving window. In the case of a multidimensional time series, the feature series stack a multifeature series with size $f_{k,m}$. Multiple features extracted from one dimension or various dimensions are grouped into a feature matrix.
**Feature Matrix ($F_{M}$):** A feature matrix with size r×(k×m), represents that each of the *k* dimensions produces *r* features. This feature matrix, which characterizes the (multidimensional) time series in statistical, temporal or spectral domains, is used to compute the self-similarity matrix.
**Self-Similarity Matrix (SSM):** A self-similarity matrix is a pairwise distance matrix between each subsequence of the time series. In this study, it is calculated by the dot product between the $F_{M}$ and its transpose. The SSM reveals several meaningful structures that indicate the presence of *events* and measures how similar segmented subsequences are. Applying the SSM, we highlight functions for the novelty segmentation and periodic segmentation process, and also introduce the similarity profiles derived from the similarity values of the SSM.
**Novelty Segmentation:** A change point event or segmentation point indicates a significant instant in time between subsequence *i* and subsequence *i* + 1, segmenting the time series. The novelty function computes such events, detailed in Section 4.3.1.
**Periodic Segmentation:** A periodic event segments the periods of a cyclic time series into subsequences. The similarity function computes these events, detailed in Section 4.3.2.
**Similarity Profiles:** A similarity profile is a time series that exhibits how similar one segmented subsequence is to all other subsequences in the time series, which helps organize the subsegments by similarity level.

### 1.2. Search Ranks

**Figure 2:**
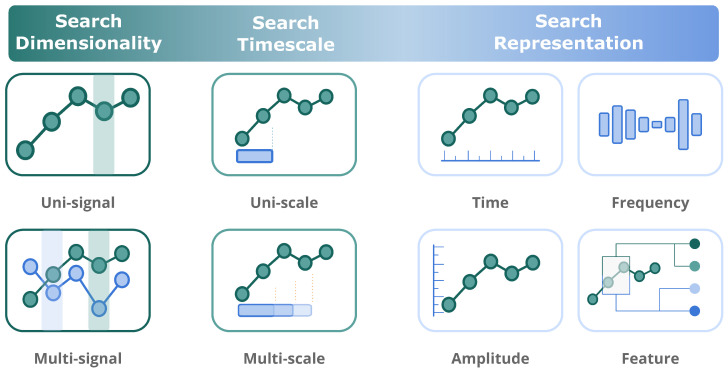
Event search in different ranks of dimensionality, timescales, and representation.

Figure 2 illustrates the search ranks of the problem formed by three layers:

- **Dimensionality**: The search can be applied to one or multiple time series. In multidimensional space, some events can coincide in several time series, while others are specified on a particular dimension. For example, some gestures produce noticeable signals on only one dimension of the three-axis ACC.
- **Timescale**: Events’ occurrence can vary from different timescales. For example, when the signal being analysed is zoomed in from hour to minute scales, some events may disappear while new events may be detected.
- **Representation**: The searchable objects can be straightforwardly the temporal nature of time series or other representations, such as frequency or other extracted features.

Besides the ranks mentioned above, the search procedure can be customized by context or target, which is highly related to the relevance given to an *event* or a *subsequence*. Types of events that are considered significant include:

- **Property change**: The change of a property, such as a change in mean or frequency, or a set of properties is greater than a threshold, e.g., 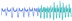.
- **Peak/Valley**: Peaks and valleys can typically be associated with significant physical changes, e.g., ECG peaks such as 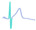.
- **Periodicity**: The starting points of each period in a periodic signal are considered relevant, e.g., Arterial Blood Pressure (ABP) periods such as 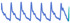.
- **Recurrent pattern**: Re-occurrences of similar *subsequences* with specific patterns should be of interest. Unlike *periodicity*, *recurrent* patterns do not have a temporal regularity, e.g., the arrhythmias found in an ECG signal such as 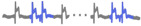.
- **Anomaly**: Highly dissimilar *subsequences* with particular patterns are of the reference value, e.g., noise in a clean signal such as 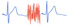.

### 1.3. Proposed Method

In order to fill as many research gaps as possible, this study started by defining the search space, considering that if the time series is transformed in the feature space, any feature’s change would be relevant. For instance, changes in the mean, standard deviation, frequency or other properties are all options worth searching for. By characterizing the signal in the feature space, we can explore changes in all feature representations. Additionally, an event should separate two different behaviours. The notion of *difference* in time series can be associated with *distance/similarity*, enabling finding segmentation points, recurrent patterns, anomalies, and periodic shapes.

Therefore, we propose an unsupervised methodology that searches for events (1) in uni- and multidimensional space, (2) with a fixed timescale and potential multi-timescale application opportunities, and (3) on an SSM computed by a feature space representation of the time series. The events to be searched are any changes in the SSM related to a segmentation point and/or a periodic event.

The proposed method’s reliability for event detection will be evidenced by considerable experiments in various type-agnostic databanks of multiple time series domains and comparisons to state-of-the-art methods. It should be highlighted that events in different datasets are extracted from the same information source, i.e., SSM.

## 2. Related Literature

### 2.1. Applications

We live in an “era of big data” [9]. As mentioned in Section 1, wearable sensors are currently available on a large scale, promoting the acquisition of massive amounts of data. Datasets of this size can no longer be handled by trivial means and call for engineers and data scientists with expertise in data mining, machine learning, and data analysis [10]. This increase in wearable usage has also been seen in industrial environments, which is motivated by the current trend of Industry 4.0 [11], promoting the use of sensors to monitor in real-time their machines for damage prevention, and their workers for occupational-related disorder prevention and productivity improvements [12,13]. Research areas such as intelligent rehabilitation [14,15,16,17,18,19], advanced sensing technologies [20], orthotics [21,22,23,24], sports science [25,26,27,28,29,30,31,32,33,34,35], activity modelling [36,37,38,39,40], exoskeletons [41,42], psychological stress classification [43], machine learning edutainment [44], surgical index monitoring [45,46], and fall detection [47,48,49], have also leveraged the power of biosignals from wearables.

In research aspects of time series analysis, biosignals produced by various types of sensors require the data science community to develop tools to extract meaningful information for the acquisition, including reporting, pattern recognition, event detection, periodic signal segmentation, and classification, among other data mining tasks [50,51]. The availability of more reliable data and practical information is more beneficial, primarily as machine learning is increasingly applied. Numerous fields could benefit from our proposed methods, including physiological event detection for healthcare (e.g., noise, sleep problems, and epilepsy), biomedical signal analysis of ECG/Electroencephalogram (EEG)/Electromyogram (EMG), climate change detection, audio-based automatic speech segmentation and recognition, motion sequence segmentation, behaviour transition detection, human activity research, feature space study, and manufacturing industries, among others.

Ultimately, data preparation is essential for data analysis and machine learning application development. After data acquisition, the effort in data processing and preparing implies challenges, an active research subject. One of the critical issues in data preparation is the lack of labelled data. Labelling data is a sensitive and time-consuming process, of which the complexity rises with the data quantity. Nevertheless, accurately labelled data are essential for data analysis and model training: In [52], the authors stated that data scientists rely solely on a small portion of the available datasets because it is too expensive to label all the data. Such a thought reflects how paramount it is to have solutions that can improve the existing data labelling strategies to reduce labour, time costs, and ground truth quality.

### 2.2. General Segmentation Approaches

Prior works in event detection focus on change point detection or segmentation, where the strategies are categorized as online versus offline, univariate versus multivariate, model-based versus non-parametric, and unsupervised versus supervised [8,53,54].

#### 2.2.1. Supervised Methods

Supervised methods include multi-class, binary and virtual classifiers optimized to detect change points [53], where the nature of the change can be provided as an additional advantage. However, supervised methods rely upon brittle training sets and class imbalance since there are more in-state sequences than change point sequences [53]. An additional problem reported by [8] is that most algorithms’ performance was validated in synthetic data, where the given nature of the application was not optimal. In response, a benchmark is available for change point detection [8], where methods can be compared on real data. This study applies this benchmark as a reference of state-of-the-art methods.

#### 2.2.2. Unsupervised Methods

Existing classic non-supervised methods in change point detection, such as the *Bayesian Online Change Point Detection* (BOCPD) [55,56], *Binary Segmentation* (BINSEG) [57], and *Segmentation Neighbourhood* (SegNeigh) [58], are witnessed to be able to perform state-of-the-art applications in various domains [8]. BOCPD is a non-supervised model-based method for change point detection that was simultaneously introduced by *Fearnhead and Liu* [56] and *Adams and MacKay* [55]. The method infers a change point based on the fact that the model parameters before and after the change point are independent. It relies on learning a joint probability distribution since the time of the most recent change point (run length) by means of a recursive message-passing algorithm. The calculated recursive probabilities will be tested to evaluate if the run length will be zero. If so, a change point is detected; if not, the run length continues to increase [8,55]. This method needs hyperparameter tuning for sound performance [8]. The BINSEG method is a greedy sequential algorithm, recursively partitioning the signal into smaller segments. The position where the signal is segmented is typically selected where the cost function is minimized. BINSEG has not been reported to cope with a multi-timescale change [8,57]. The available repository [8] collecting the implementation of some offline methods [54] above lacks a visual output that can provide users with the location of the change points. In this work, we used the benchmark dataset available in this repository to compare the performance of the proposed solution with the mentioned methods.

Window-based segmentation, typically relying on a sliding window that is divided into two smaller windows based on the comparison using a cost function, can apply to real data domains [54]. Another approach, called *Fast Low-cost Online Semantic Segmentation* (FLOSS) [59,60], searches regime changes based on the nearest neighbours of subsequences, which allows the similarity comparison between segments for the segmentation and summarization of long-term time series.

### 2.3. Biosignals’ Segmentation Approaches

Some general approaches mentioned in Section 2.2.1 and Section 2.2.2 do not necessarily work well for biosignal segmentation tasks. Emerging works focus specifically on biosignal segmentation, e.g., applying neural networks (NN) to ECG signals. In [61], an NN with transfer learning was used for the segmentation of periodic biosignals (motion and ECG). Convolutional NN has also been found for ECG segmentation. In [62], a convolutional NN on a binary classification task (*heartbeat* or *not a heartbeat*) was proposed, while Aman Malali et al. put forward a convolutional long short-term memory (LSTM) NN for the same task [63]. Last but not least, *Viktor Moskalenko* et al. used a UNet-like full convolutional NN for the ECG signal segmentation of P and T waves, as well as the QRS complex [64]. Without model training, the ECG segmentation can also be solved through a subsequence search in the context of a carefully selected query pattern [65].

Wearable technology has also improved the field of gait analysis, increasing interest in gait event detection [66]. Recently, machine learning approaches have been found for gait segmentation related to Parkinson’s disease [66], and hidden Markov models (HMM) were also used for the same purposes [67]. Gait event detection methods based on rapid positive changes in Gyroscope (GYR) data were employed for rehabilitation research [68,69]. The work from *Matteo* et al. shows the ability of deep learning techniques to improve gait segmentation [66]. Traditional signal processing methods, such as the integral of the signal envelope, can be applied to EMG signal segmentation for gait analysis [70].

Biosignals’ segmentation also facilitates medical research. For the study of sleep staging segmentation, *Mathias Perslev* et al. introduced a fully convolutional network [71]. A square-root velocity function to segment periodic data for posterior alignment and the statistical analysis helped disease classification based on the *Karcher* mean [72].

### 2.4. Segmentation with the Self-Similarity Matrix (SSM)

The SSM has been used for segmentation in the audio domain, based on a feature representation of the audio signal [73]. The advantage of the SSM is that it provides a considerable amount of information for a specific timescale. This study promotes SSM concepts and applications from the audio domain to other time series domains. The proposed method can detect events with context, associating the estimated events with patterns, (dis)similarities, periodicity and novelty, and a possible extension is the task of summarization. The search mechanism is primitively based on a specific timescale and can evolve recursively to perform multi-timescale searches.

## 3. Datasets

In order to test and validate the proposed method, we applied public datasets with segmentation requirements. As a multimodal, complex patterned, and versatile type of time series signals, sensor-based biosignals are the experimental target of this study: inertial signals for motion in the domain of HAR, EMG for onset/offset detection and ECG for noise detection. The datasets and their sources are described below.

### 3.1. Dataset 1—HAR

Each participant of the 30-subject dataset [74,75] was wearing a *Samsung Galaxy A2* smartphone on his/her waist while performing the following activities: *(1) Walking, (2) Walking Upstairs, (3) Walking Downstairs, (4) Sitting, (5) Standing and (6) Laying*. Each activity was performed for approximately 60 s and labelled. The device records the internal ACC and GYR data at a constant rate of 50 Hz.

The ACC of the dataset was used to search for segmentation points on the signal. The usage of both sensors was not possible because the ACC and GYR signals are not synchronized with each other. The absence of average variation makes the switch between the static poses (4)–(6) listed above not evident for GYR. Therefore, we only used the ACC channels in this study.

Generally, ACC is recognized as one of the most helpful wearable sensors in multimodal biosignal-based HAR. For instance, [76] demonstrates that ACC’s HAR performance outperforms other kinds of sensors. Each activity label uses the same timestamp as the corresponding signals. We defined the ground truth for segmentation borders as the switch of labels.

### 3.2. Dataset 2—ECG1

The dataset [77,78] comprehends 12 half-hour ECG acquisitions and 3 half-hour collections of noise typical in ambulatory ECG recordings. The noise recordings were collected from physically active volunteers using standard ECG trackers, leads, and electrodes. The three noise records were assembled from the recordings by selecting intervals containing predominantly baseline wander (in record “bw”), muscle (EMG) artefact (in record “ma”), and electrode motion artefact (in record “em”). Two selected clean ECG signals were noised with different Signal-to-Noise-Ratio (SNR).

This dataset was used in the context of change point detection to validate the proposed method for estimating transitions to and from signal sections with added noise. The subsequences with standardized noise, annotated by an expert in time series data mining, were applied as our experimental ground truth.

### 3.3. Dataset 3—ECG2

The dataset [78,79] for studying ECG’s motion artefacts and sparsity encloses short-duration ECG signals at 500 Hz recorded from a healthy 25-year-old male performing the physical activities of standing, walking, and single jumping.

This dataset was used in the context of change point detection to validate the proposed method for estimating transitions to and from sections with noise added due to a jump.

### 3.4. Dataset 4—EMG

A *Myo Thalmic* bracelet worn on the user’s forearm was applied for the 36-subject dataset [80]. The bracelet is equipped with eight sensors equally spaced around the forearm that simultaneously acquire EMG signals at 200 Hz. Each participant performed two recording series, each consisting of six three-second basic gestures with a three-second pause between each gesture pair.

In the context of change point detection, this dataset helped validate the proposed method for estimating transitions between the activation and relaxation of the muscular activity. Each activity label uses the same timestamp as the corresponding signals. We defined the ground truth for segmentation borders as the switch of labels.

### 3.5. Dataset 5—CPDBenchmark

For an objective evaluation, we also compared the proposed method with existing approaches on a change point detection benchmark [8], comprising several time series from real-domain contexts. The repository was built by the Alan Turing Institute for the evaluation of change point detection algorithms.

This dataset has ground truth events for each time series. In addition, the available performance of several existing approaches was compared to the proposed method’s results on the same time series. Our proposed method is also foreseen to be competent in other sorts of unidimensional or multidimensional time series, and related studies are on the agenda.

### 3.6. Dataset 6—BVP (for Illustrative Instances)

In the dataset [81], ten subjects’ slow tilt, rapid tilt and standing-up activities were monitored and recorded with ECG and Blood Volume Pressure (BVP) to investigate how the two physiological signals respond to the angular changes during the activities [78,81].

The dataset’s BVP channel was used as an example to demonstrate the proposed algorithm’s capability in detecting pattern-based physiological changes in a distinctive research instance. The ground truth of the changes is marked by the angular signal, suggesting the moments a tilt or standing-up activity occurred.

### 3.7. Dataset 7—ECG Pulsus Paradoxus (for Illustrative Instances)

The signal used was extracted from the dataset available in the *UCR Semantic Segmentation Benchmark* [60]. The signal represents an ECG recorded from a patient who had an onset of *pulsus paradoxus* [82,83].

The signal, with a regime change at the 10,000${}^{\mathrm{th}}$ sample, was used as an illustrative application scenario for the proposed method.

## 4. Method

**Figure 3:**
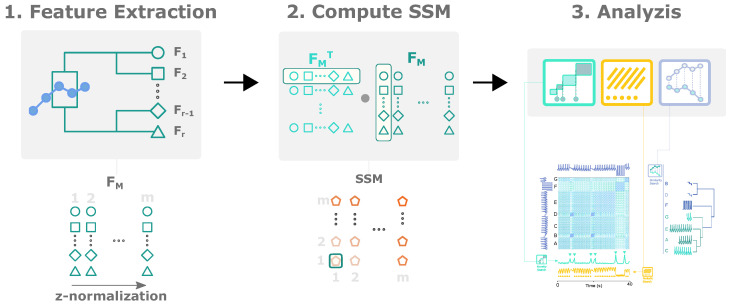
A step-by-step flowchart for calculating and analysing the SSM. The signal-based calculation requires input parameters of the window size *w* and the overlapping percentage *o* to fulfil the first-stage feature extraction. Features are extracted on each subsequence $\left(sT_{1},sT_{2},\ldots ,sT_{N}\right)$, where *N* is the total number of windows. *K* features are extracted from window *i* ($sT_{i}$: $f_{i_{1}},f_{i_{2}},\ldots ,f_{i_{K}}$). Different features are associated with different shapes (◯,□,⋄ and Δ) in the figures. The features can be extracted on an *M*-variable record and each feature is positioned as a row on the $F_{M}$ for the SSM computation.

The extraction of relevant events from time series starts by computing the SSM. As explained in Section 1.1, the SSM has relevant structural information to retrieve *events*, namely *blocks*, *paths* and *similarity profiles*. Figure 3 summarizes the calculation steps for the SSM.

### 4.1. Feature Extraction

The structural information on the SSM reflects how informative the feature set can translate the signal’s changes and disruptions. Behavioural changes may be related to a variate set of features. As a feature can be sensitive to a particular type of change, the set of features should be diverse to identify a multivariate set of events and be agnostic to various signal types. We turned to the available features from the *Time Series Feature Extraction Library* (TSFEL) [84] for *new*, which has been proven effective and efficient in previous work on multimodal biosignal processing [36,85,86] and other research fields [87,88,89,90,91]. We selected over 50% of all TSFEL features in the statistical, temporal, and frequency domains with relatively lower computational costs, as listed in Table A1 in Appendix A, regarding our proposed method’s high calculation resource consumption.

The features are extracted with a moving window with size *w*, specified by the user, with an overlap percentage *o*. The selection of the two sizes significantly influences the results: *w* defines the timescale at which features are extracted so that the wider the window, the more *zoomed-out* the search will be. The second parameter defines the pixel resolution of the resulting feature series, increasing the amount of information with a larger overlap.

The extracted features are grouped into a feature matrix ($F_{M}$), where the rows represent a feature series and the columns correspond to all subsequences. In the multidimensional case, *r* features extracted from each of the *k* dimensions are ordered in the $F_{M}$ as rows, forming r×k elements in each row, as illustrated in Figure 3.

Each feature series (rows of the $F_{M}$) is z-normalized for a more balanced contribution to characterizing the signal. A further normalization is applied to the feature vector (columns of the $F_{M}$), which optimizes the cosine similarity computation between feature vectors by simply adding the dot product to calculate the SSM.

### 4.2. Feature-Based SSM

After grouping all the features extracted, the next stage is to apply a similarity measure to the feature space and compute the SSM. This process consists in comparing each *subsequence* with all the other *subsequences*. Since each column of the $F_{M}$ is each subsequence’s feature characterization in the entire feature set, the SSM, i.e., the comparison between segments, is obtained by calculating the dot product between the z-normalized transposed $F_{M}$ and itself:

(1) $$SSM=F_{M}^{T}\cdot F_{M}.$$

The dot product scores the similarity based on the subsequence’s feature values. Cells of the SSM with higher similarity scores indicate that the corresponding *subsequences* have similar feature values [3,4]. As a result, the SSM provides rich visual information, highlighting structures that describe the signal’s morphological behaviour over time and structure, such as blocks and paths.

**Figure 4:**
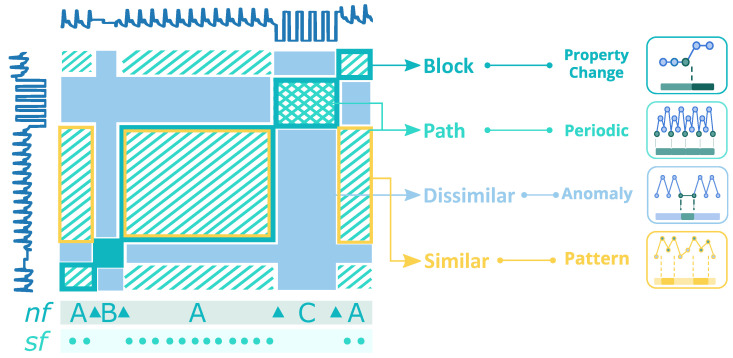
The informative structures of an ABP signal’s SSM. The three main structures are highlighted in the simplified illustration: A—the homogeneous segments corresponding to periods in the ABP signal; B—the homogeneous segment representing missing data; C—the homogeneous segment cueing sensor detachment. The “blocks” in the figure accentuate homogeneous behaviour, while the paths in the figure depict periodicity in the segment. Segment C has a cross pattern, which symbolizes periodicity and symmetry. nf: novelty function; sf: similarity function; Δ: change points separating blocks A, B and C.

In Figure 4, the main structures are illustrated and highlighted in an example of an *SSM* [3] computed from an ABP signal, where the main structures are *blocks* and *paths*. Our proposed method utilizes the resulting main structures to extract the desired information.

*Paths* show recurrence of patterns, which indicates the morphological matching between corresponding *subsequences*. Circles in the *sf* layer exhibit when the paths start. The *cross-pattern* in *block* C means that the *subsequences* are periodic and symmetric.

Differently, *blocks* are square-shaped structures of homogeneous areas in the SSM, translated as constant behaviour in the time series. The change between block structures along the main diagonal displays a relevant change in morphology and behaviour in the time series. In Figure 4, the SSM is segmented into several blocks on layer *nf*, for which the Δs mark the change points that separate blocks A, B and C. Besides *paths* and *blocks*, the SSM provides similarity measures between *subsequences*, which can be used to spotlight (dis)similar segments, such as anomalies, motifs or cycles. Several strategies were applied to the SSM to extract the mentioned information.

### 4.3. Information Retrieval

The SSM is a powerful visual tool per se, exposing relevant information that a raw observation could miss. Automatic discovery of information of interest will increase the SSM’s practicability and versatility, for which three approaches for information retrieval on the SSM are put forward: (1) novelty search of *block* transitions, (2) periodic pattern search of *paths*, and (3) similarity profiles of *subsequences*.

#### 4.3.1. Novelty Search

The search for *novelty* is inspired by a method used in musical structure analysis [92], which is computed with the help of the *libfmp Python* package [93]. The process involves searching for transitions between *blocks* using a moving chequerboard square matrix, resulting in a one-dimensional function: the *novelty function*.

**Figure 5:**
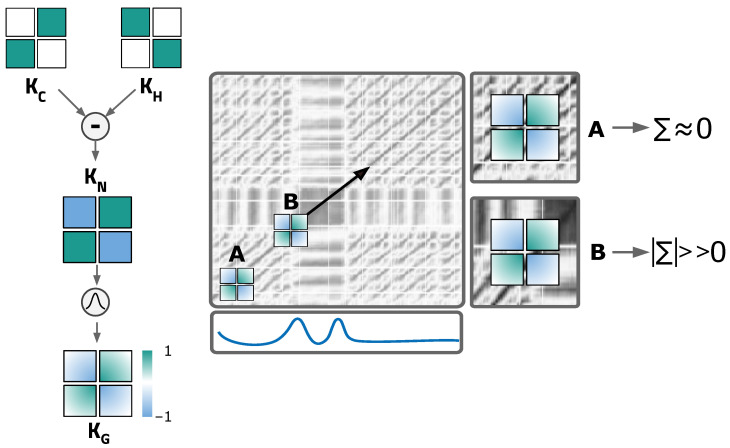
(**Left**): description of the matrix (kernel) used to compute the *novelty function*, based on the works of *Mueller* et al. [2,73]. The chequerboard pattern of the kernel $K_{N}$ is achieved by combining the kernel $K_{H}$ (homogeneity measure) and $K_{C}$ (cross-similarity measure). Combined with a Gaussian function, the $K_{G}$ is obtained; (**right**): the process to compute the novelty function based on the works of [2,73,94]. Kernel $K_{G}$ slides along the diagonal of the SSM to compute the *novelty function* presented as the bottom sub-plot. Positions A and B point to the effect of block transitions on the *novelty function*.

As shown in Figure 5, *block* transitions along the diagonal are represented by a chequerboard pattern. Such patterns can be detected by correlating a standard chequerboard matrix with the diagonal of the SSM, for which a sliding squared matrix, designated *kernel*, is used. The kernel incorporates a Gaussian function with a smoothing factor. The kernel $K_{N}$ combines two different square matrices: $K_{H}$ and $K_{C}$. $K_{H}$ is responsible for identifying the homogeneity of the SSM on each side of the centre – the more homogeneous the pattern is, the higher the corresponding values will be. $K_{C}$ measures the cross-similarity level. Therefore, when sliding the kernel $K_{N}$ along the diagonal, a higher correlation value will be returned when it reaches a segment of the SSM with a similar chequerboard pattern. The result is the mentioned novelty function [2,73,94].

In position A of Figure 5 (right), due to the high homogeneity, the kernel returns a value approaching 0 when summing the product between it and the section of the SSM it overlaps. In contrast, the kernel in position B reaches a segment with low cross-similarity and high diagonal similarity, which results in high correlation values with a chequerboard pattern. Therefore, high *novelty function* values are witnessed in these transition segments [2,73,94].

Each section of the kernel has the same size, L∈N, and D=2×L+1 configures the total kernel size. The kernel has an odd size to adapt zero values in centred points, and a total size of D×D. $K_{N}$ is defined by [2,73]:

(2) $$K_{N}\left(i,j\right)=sign\left(a_{i}\right)\cdot sign\left(b_{j}\right),$$

where a,b∈[−L:L] and sign represent the sign function (1, 0 or −1). A radially symmetric Gaussian function is used to smooth the Kernel [2,73]:

(3) $$\phi \left(p,u\right)=\exp(-\frac{1}{2L\sigma^{2}}\left(p^{2}+u^{2}\right)),$$

where σ is the standard deviation, equal for both *x* and *y* dimensions of the matrix, *L* the size of each kernel’s section, and *p* and *u* the position in the *x* and *y* dimensions, respectively. The kernel $K_{G}$ is computed by point-wise multiplication with the Gaussian function:

(4) $$K_{G}=\phi \cdot K_{N}.$$

The *novelty function* $n_{f}$ is calculated by correlating the kernel with the diagonal of the matrix:

(5) $$n_{f}\left(m\right)=\sum_{i,j=0}^{2L+1}K_{G}\left(a_{i},b_{j}\right)SSM\left(m+a_{i},m+b_{j}\right),$$

being the sample of the novelty function m∈[0−N] and a,b∈[−L:L]. The change point events are represented by local maxima (peaks) in the *novelty function*, which can be detected by standard peak-finding strategies.

#### 4.3.2. Periodic Search

As aforementioned, *paths* indicate the presence of similarity and reoccurring patterns can be visualized on the SSM. The *path*’s start point punches where the period of the pattern begins. In order to find the periodicity, we compute the similarity function $s_{f}$ by summing the values of the symmetric SSM column-wise or, equally, row-wise. Each element of the sf is calculated by

(6) $$sf\left(x\right)=\sum_{i=0}^{m}SSM_{ix},$$

where *i* is the column position for the sum, $sf_{j}$ is the sample of the function at position *j* and *m* is the feature-series size. As segments with similar morphology will be similarly described by the extracted features, the columns will have a similar representation, resulting in similar values on the *sf*. The similarity function will enhance such behaviour when facing periodic series. The identification of events related to the periodicity of a time series is then feasible by searching for local minima (valleys) on the similarity function.

Although not validated in this work, an additional application of the similarity function should be outlooked. Considering that each sample of the sf is an average similarity of a subsequence to all other subsequences, it is possible to find *anomalies*. Regarding an *anomaly* as a subsequence highly unique and different from all the rest of the time series, its average similarity to all the other subsequences should have a low value.

#### 4.3.3. Similarity Profiles

The principal elements, *blocks* and *paths*, are the information basis for segmenting the time series. Besides, SSM also provides pairwise similarity values between all *subsequences* of the time series, an important measure that can be used for clustering and *motifs*/*discords* discovery. The similarity profiles exploit the similarity values of the SSM to facilitate *subsequences* comparison. A similarity profile charts the similarity values of a *subsequence* (one column/row of the SSM) to all the other *subsequences*. The higher the values, the more similar the *subsequences* are. In addition to the *subsequences*’ comparison, the similarity profile can also compare between entire segments of the signal. For instance, all three *A*-segments highlighted in Figure 4, whose profiles are highly similar despite the different sizes.

Although the segment comparison could be directly based on the region of the SSM delimited by two *subsequences*, we propose a more effective measure of two segments’ similarity/difference according to their similarities/differences to all the other *subsequences*. A *similarity profile* $P_{s}\left(c\right)$ of a segment is computed as the column(row)-wise average similarity values of the region delimited by the segment being profiled (size *l*), and all the other *subsequences* of the time series (size *m*):

(7) $$P_{s}\left(c\right)=\frac{\Sigma_{i=0}^{l}SSM\left(i,c\right)}{l}.$$

The *similarity profile* is computed column(row)-wise. Each column(row) c(r) is the average similarity value between the reference segment and the segment corresponding to *c*. The reasoning is that similar segments should have closer *similarity profiles*. Since the profiles have the same size, they can be compared by certain distance measures, such as the Euclidean Distance (ED), to form clusters, an automatic clustering solution based on the segments generated by the *novelty* and *similarity* functions.

## 5. Experimental Analysis, Validation, and Discussion

### 5.1. Illustrative Evaluation in Various Application Scenarios

Experiments from multiple domains were carried on to validate the practicability and universality of the process to represent the time series into a feature-based SSM and the method of retrieving information from the SSM.

#### 5.1.1. Acceleration Signals in Human Activity Domain

**Figure 6:**
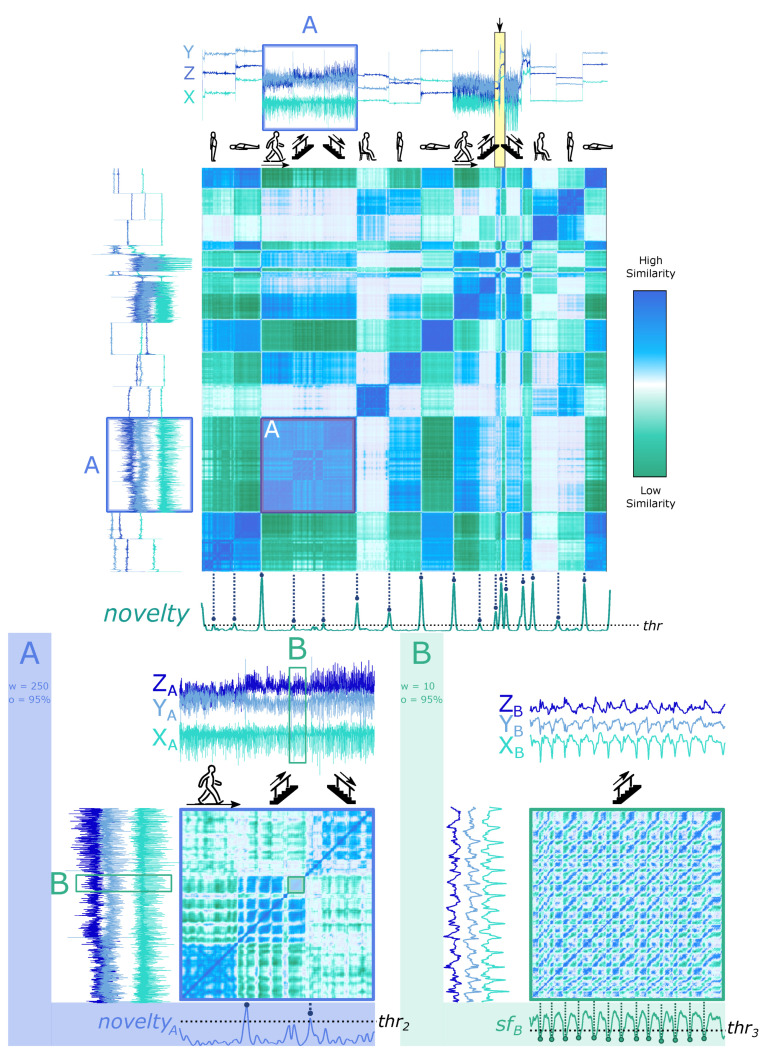
An SSM-based novelty search strategy to detect segmentation events on a signal piece from Dataset 1 (HAR, see Section 3.1). (**Top**): $window_{size}$ = 250 samples, $kernel_{size}$ = 45 samples, and overlap = 95% on the activity sequence $Standing\overset{}{\to }Laying\overset{}{\to }Walking\overset{}{\to }Upstairs\overset{}{\to }Downstairs\overset{}{\to }Sitting\overset{}{\to }Standing\overset{}{\to }Laying\overset{}{\to }Walking\overset{}{\to }Upstairs\overset{}{\to }Downstairs\overset{}{\to }Sitting\overset{}{\to }Standing\overset{}{\to }Laying$. The novelty function is presented and **peaks** are aligned with ground truth events, represented as the dashed line and circles; (**bottom left**): signal change point detection on segment *A* with a size of 5000 samples, an overlap of 75%, and a kernel size of 25 samples. The novelty function is displayed and **peaks** are aligned with ground truth events, represented as the dashed line and circles; (**bottom right**): further zooming in with a window size of 10 samples and an overlap of 95%, to reveal more periodic details of segment *B*. The similarity function is presented and **valleys** are aligned with ground truth events, represented as the dashed line and circles.

Accelerometers are usually considered one of the most effective sensors for wearable-based HAR [76,95]. Figure 6 (top) exemplifies the SSM’s usage on a record of Dataset 1 (HAR, see Section 3.1), where the data of all three ACC axes are applied. The SSM was computed using a 250-sample window size and a 95% overlap. Along the diagonal, the novelty function generates block-wise references for estimating activity transition using a 45-sample kernel.

We can identify in Figure 6 (top) that the detected segmentation points match the activity transitions. Although all transitions are visible on the novelty function, the transitions between similar activity patterns in the walking category (straightforward, upstairs and downstairs) are more challenging to differentiate, as block *A* suggests, which is plausible since the properties of these segments are morphologically similar.

The proposed unsupervised method automatically and sensitively detects any significant change in properties. As can be found in the yellow-marked part in Figure 6 (top), the period in which the subject was performing the *Upstairs* activity is affected by other changes in the time series. These are significant and also correspond to *block* transitions, which are also evident in the novelty function.

When *zooming* the SSM into segment *A* in Figure 6 (top), the three activities in the walking category can be effortlessly segmented based on the change points revealed by chequerboard patterns, as the two most prominent peaks in the corresponding novelty function pinpoint (see Figure 6 (bottom left)). In addition, it is noticeable that the matrix segments related to *Upstairs/Downstairs* are also segmentable into smaller *blocks*. As the information is not available in the dataset description, we believe they are a flight of stairs.

Questions may arise at this point. Why is the signal periodicity of the three walking activities not evident in Figure 6 (bottom left)? The reason is that the window size used to compute the SSM is relatively large. If features are extracted with a smaller window size closer to the walking period, the *paths* delineating the pattern recurrence will be visible. Figure 6 (bottom right) shows the SSM built from segment *B* of the original time series, with a window size of 10 samples and an overlap of 95%. The *paths* in the matrix enable the periodicity detection with the similarity function $sf_{B}$.

#### 5.1.2. Arterial Blood Pressure (ABP) Signals in Posture Recognition Domain

Many biomedical signals, such as ECG, ABP, and Respiratory Inductance Pletismography (RESP), contain retrievable structural information such as periodicities. Meanwhile, unexpected changes may occur during the acquisition due to physiological responses, medical disorders, or sensing problems such as noise, interferences, artefacts, and electrode detachment. We visualize two examples of physiological changes in different types of periodic signals.

**Figure 7:**
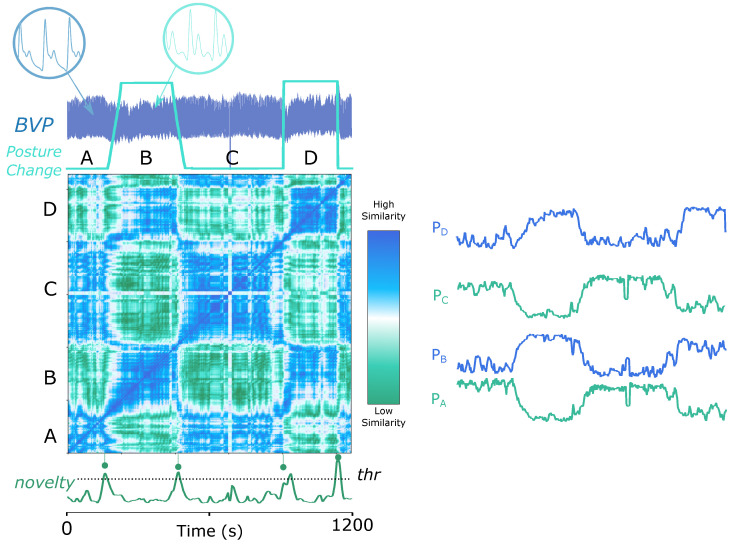

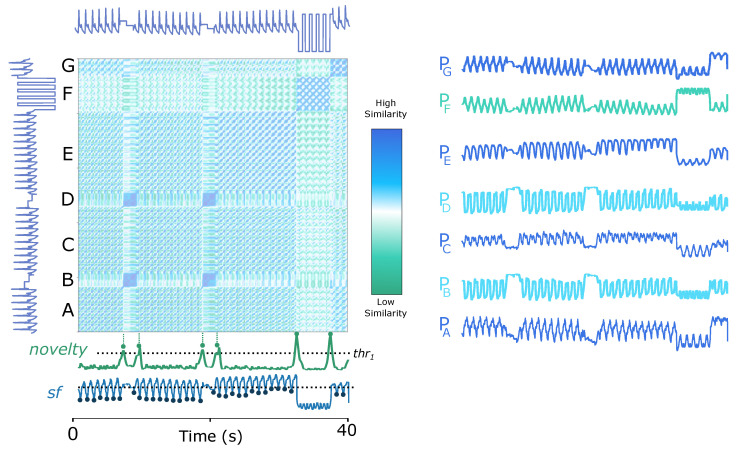
Novelty and similarity search on an ABP signal from Dataset 6 (BVP, see Section 3.6). (**Top**): a window size of 5000 samples, an overlap of 95%, and a kernel size of 200 samples. The trapezoidal and the square wave mark the ground truth of slow and fast postural transitions. Similarity profiles $P_{A}-P_{D}$ show how similar each segment (A−D) are. For instance, $P_{D}$ is more similar to $P_{B}$. (**Bottom**): the first 10,000 samples, with a window size of 250 samples, an overlap of 95% and a kernel size of 200 samples. The right parts of the top and bottom subfigures plot the corresponding similarity profiles for each subsequence segmented by the novelty function. In both figures, the novelty function is displayed and **peaks** are aligned with ground truth events, represented as dashed lines and circles. The bottom plot also shows the similarity function (*sf*) with circles representing the ground truth of periods. In addition, similarity profiles $P_{A}-P_{G}$, show how similar are each segment resulting from the novelty function. For instance, segment B is more similar to segment D, and this is verified by $P_{D}$ being more similar to $P_{B}$.

The ABP signal can vary due to postural changes, as an available experiment at *Physionet* confirms [78,81]. Figure 7 (top) shows the process of segmenting the ABP signal based on postural changes, where the trapezoidal and the square wave tag the ground truth of slow and fast postural transitions. The proposed strategy well perceives the change points. Observably, the shape of the raw ABP signal in each regime is undistinguishable through the naked eye. Therefore, it is constructive to rely solely on the signal itself to implement postural change detection. It is important to point out that the periodicity of the signal is not visible on the matrix because the features were extracted with a window size of 5000 samples, which is much larger than the period size. A smaller window size of 250 samples in the current scenario allows periodic segmentation, as Figure 7 (bottom) illustrates, where the SSM is computed on the first 10,000 samples of Dataset 7 (ECG Pulsus Paradoxus, see Section 3.7). The resulting *similarity* function gives prominence to the periodic nature of the ABP signal.

The SSM of Figure 7 (top) also shows which segments are similar to each other. The blue-coloured parts in the matrix indicate high similarity, presenting that segments from the same posture are more similar than between different postures. For further illustrative purposes, we computed the similarity profiles of each segment as if segmented by the *novelty* function, which evidences that the corresponding sections could be well clustered based on the similarity profiles ($P_{A}=P_{C}$ and $P_{B}=P_{D}$). In the same way, the similarity profiles in Figure 7 (bottom) examine the similarity between segmented *subsequences*. Profiles with a similar shape can be grouped together ($P_{A}=P_{C}=P_{E}=P_{G}$ and $P_{B}=P_{D}$), which can be applied to automatic clustering, as exhibited in Figure 1.

#### 5.1.3. Electrocardiography (ECG) Signals in Biomedical Domain

**Figure 8:**
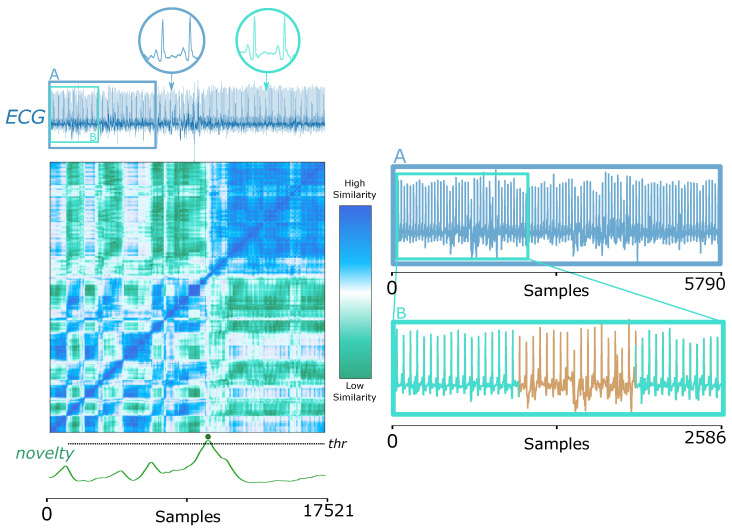
An ECG signal with a *pulsus paradoxus* condition starting at the 10,000th sample from Dataset 7 (ECG Pulsus Paradoxus, see Section 3.7). (**Left**): the SSM diagnoses two modes in the signal, whose patterns are zoomed in the circle thumbnails, respectively; (**right**): zooming parts of the original signal can verify SSM’s ability of automatic ECG pattern change detection and contribution to segmentation. The novelty function is presented, and the peak is aligned with the ground truth event, represented as a circle.

Another widely used biomedical signal, ECG, also testifies to the feasibility of our proposed method. The ECG signal in Figure 8 (left) displays the presence of the condition called *pulsus paradoxus*, an exaggerated fall (>10 mmHg) in the subject’s blood pressure during inspiration [83], which can also occur when the patient changes sleeping posture after heart surgery [60], as the following example relates. Similar to the ABP signal elucidated in Section 3.6, the human eye hardly perceives the change points in ECG signals. Once again, our proposed strategy shows strength.

In addition to the novelty detection, segment A previous to the *pulsus paradoxus* occurrence can be partially detailed again to reveal minor changes due to additional noise, verifying SSM’s sensitivity to structural changes, as Figure 8 (right) imparts.

#### 5.1.4. Single Channel versus Multidimensionality Application in Multi-Sensor Scenarios

**Figure 9:**
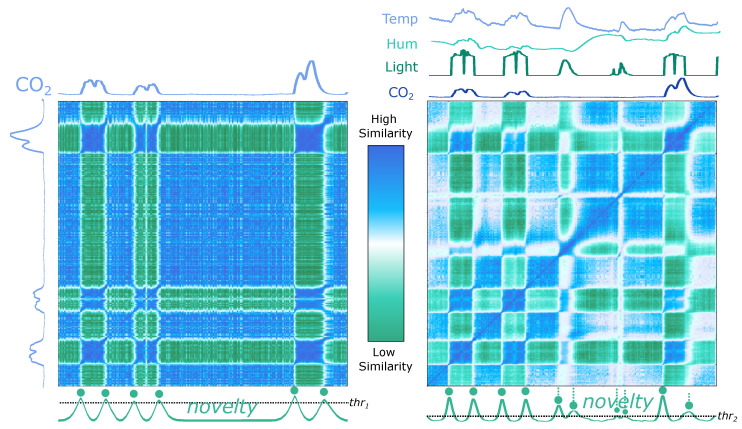
The proposed method was applied to the *Occupancy* record of Dataset 5 (CPDBenchmark, see Section 3.5). (**Left**): calculations on the separate $CO_{2}$ time series only; (**right**): calculations performed by extracting features on the complete four time series. The novelty function is presented and *peaks* are aligned with ground truth events, represented as the dashed line and circles.

The proposed method accepts both single- and multidimensional records. The difference regards the number of features extracted. As compared in Figure 9, the same set of features is extracted from each time series to build the $F_{M}$. Using a single or several time series of a multidimensional record is an option, depending on the purpose. In some cases, the use of non-complete dimensions may miss relevant events, as Figure 9 instances the record “Occupancy” from Dataset 5 (CPDBenchmark, see Section 3.5).

The record is a multidimensional time series that measures room occupancy based on temperature, humidity, light, and $CO_{2}$. By comparing the signals and formations in the left and the right parts of Figure 9, it can be understood that some events can be detected using the $CO_{2}$ series exclusively, but some are missed.

### 5.2. Statistical Performance Evaluation

In order to evaluate the performance of our proposed method with biosignals as well as in general scenarios, we applied the algorithm to all datasets introduced in Section 3. The evaluation was divided into *biosignals-related applications* and the general change point detection benchmark (Dataset 5). The biosignals experiments are associated with public datasets 1–4 from Physionet, the *UCI Machine Learning Repository* and the *UCR Semantic Segmentation Benchmark*, involving different contexts (HAR, hand posture, and noise detection) and sensor types (ACC, EMG and ECG).

#### 5.2.1. Metrics for Quantitative Evaluation

The quantitative evaluation on biosignals’ public datasets was made by accumulating true positive (TP), false positive (FP), and false negative (FN) values with a tolerance zone around the ground truth events. The applied reasonable tolerance was the ground truth wrapped by a window size of the SSM computation, inside which a detected event was counted as a TP. The case that no estimated event was found inside the tolerance band was considered an FN. An estimated event outside the tolerance or duplicating an already counted TP was regarded as an FP. The F1-score, based on the precision and recall values, was calculated from TP, FN, and FP values, following Equations (8)–(10):

(8) $$Precision\;\left(P\right)=\frac{TP}{TP+FP}$$

(9) $$Recall\;\left(R\right)=\frac{TP}{TP+FN}$$

(10) $$F1-score\;\left(F1\right)=\frac{2}{\frac{1}{P}+\frac{1}{R}}=2\cdot \frac{P\cdot R}{P+R}$$

**Figure 10:**
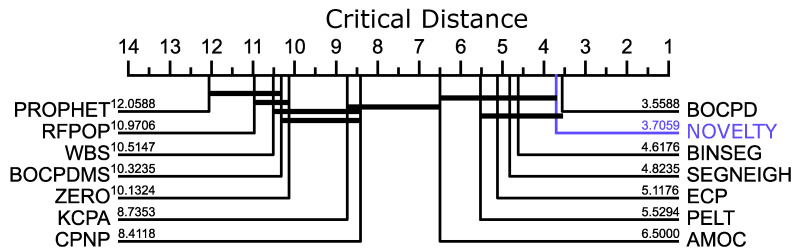
Critical distance diagram comparing the methods used in [8] (except *RBOCPDMS*) and the *novelty function* on Dataset 5 (CPDBenchmark, see Section 3.5). The performance measure corresponds to the F1-score for all single-dimension datasets of the benchmark, except for the ones identified in Table 3 with a grey background. A thick horizontal line groups a set of classifiers that are not significantly different in the statistical test [96].

In Section 5.2.2 and Section 5.2.3, we present two evaluation layers: (1) evaluation of biosignals’ segmentation and (2) benchmark evaluation. On (1), the performance of our proposed method was compared with existing approaches available on the *Python* library *ruptures*, namely the window-based segmentation (WS) and the binary segmentation (BS) [54] based on the F1-score. The benchmark evaluation referred to the best score obtained from the state-of-the-art methods available on the repository [8]. The evaluation procedure to detect TP, FP, and FN was the one followed on [8]. In addition, we also compare the F1-scores of all methods with a critical distance plot in Figure 10. The plot associates statistical tests over the F1-scores of each method. The test evaluates whether the performance difference is significant (critical difference) or not. In this work, we borrow an implemented critical difference method of [96] that uses the *Wilcoxon–Holm* test [97], which counteracts the problem of multiple comparisons and calculates pairwise significance between all methods evaluated.

The method has been computed in the same conditions and followed the same procedure for all datasets’ records. The features used were the same for each record (see Appendix A), varying the timescale parameter, the overlap size of the sliding window, and the kernel size. The peak detection strategy based on a threshold mechanism is the same for all records, while the threshold value varies from record to record. Results for publicly available datasets are listed in Table 1 and Table 2, and Table 3 expands the performance by F1-scores in detecting the change point events.

#### 5.2.2. Biosignals’ Segmentation

**Figure A1:**
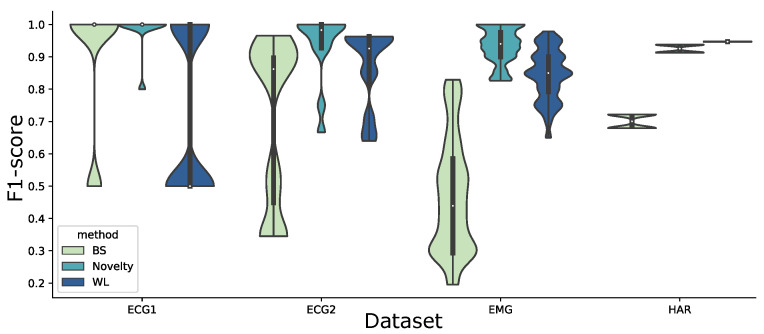
F1-scores’ distribution for three segmentation methods on Datasets 1–4. WS: window-based segmentation; BS: binary segmentation; Novelty: the proposed novelty function-based segmentation.

The illustrative examples provided in Section 5.1 corroborate the proposed method’s capability in segmenting real, complex, and multimodal biosignals datasets. As Table 1 conveys, an overall macro-averaged 0.94 F1-score is achieved, while the competitors’ overall F1-scores are 0.84 (WS), and 0.69 (BS), respectively. Table 2 broadcasts the F1-scores of our method on the matches against the other two methods in the form of “Win/Draw/Lose”, announcing that it has, in most cases of Datasets 3 (ECG2, see Section 3.3) and 4 (EMG, see Section 3.4), higher F1-score, while loses in Dataset 1 (HAR, see Section 3.1) and draws in most cases for Dataset 2 (ECG1, see Section 3.2). Overall, it has more wins in three of the four tested datasets. Table A2, Table A3, Table A4 and Table A5 in Appendix A detail the parameters of window sizes, kernel sizes, and thresholds applied to the signals in each dataset, as well as the obtained F1-scores. An intuitive graphical comparison can be found in Figure A1, which plots the distribution of F1-scores in each dataset.

For Datasets 1, 3 and 4 (HAR, ECG2, and EMG, see Section 3.1 and Section 3.4 respectively), the window-based methods, *novelty* and WS, performed much better than the BS method, mainly because the sliding window algorithm with a full set of features comprehensively characterizes changes in the signal. The standard WS uses cost functions searching for mean/variance value changes in the signal, which achieves a high F1-score in Dataset 1 (HAR, see Section 3.1), even identifying transitions between dynamic activities, such as *Walking*/*Upstairs*. Our proposed method had a similar performance with a worse count in FP values, where the added features did not improve the segmentation performance. In contrast, our method, complemented by additional features, had a much better performance than the WS method in Dataset 4 (EMG, see Section 3.4). Adding features enabled a more robust and sensitive detection of pattern changes, although it missed some changes between similar patterns, such as *Walking* and *Upstairs/Downstairs* in Dataset 1 (HAR, see Section 3.1), which are the primary source of the FN value. Similar to the FN values, the FP values of our proposed method are mostly superior to other methods. It nonetheless leaves room for discussion. Some events are not marked as changes in specific activities, but the signal pattern actually changes. For example, Figure 6 (bottom) exposes pattern changes during an *Upstairs*/*Downstairs* activity unlabelled in the ground truth, possibly due to a flight of stairs. The novelty function is sensitive to such pattern changes, which inevitably contributes to the FP values during the comparison with the ground truth. Considering the good performance of both methods, further research should be made in other HAR domains to understand the differences in performance between them better.

Specifically for ECG signals, our proposed method shows its capability on both Datasets 2 (ECG1, see Section 3.2) and 3 (ECG2, see Section 3.3). Although ECG-based jump artefact detection is fundamental, the WS method could not find the segmentation borders, while the BS method worked better. In Dataset 3 (ECG2, see Section 3.3), the same ECG signal was noised with different SNR levels to form a new set of resultant signals. Overall, our proposed method was able to detect the changes between noisy segments and clean segments with noise down to 12 dB. At 6 dB, the proposed method achieved an F1-score of 0.67 with references of 0.64 (WS), and 0.34 (BS).

#### 5.2.3. Segmentation Benchmark

In order to compare the proposed method with other state-of-the-art approaches, we used a benchmark provided by the Alan Turing Institute [8] (Dataset 5—CPDBenchmark, see Section 3.5). The performance was evaluated by change point event detection in each time series available, summarized in Figure 10, where each referenced method applied its best score in the benchmark (see Table 3 and Table A6).

As unfolded in Figure 10, the critical distance diagram ranks the proposed method second, suggesting no significant difference in performance among methods that only work in uni-dimensional datasets. The global average F1 measure of the proposed method is 0.87 for both uni and multidimensional datasets. Overall, the proposed method had a total of 16 higher F1-scores than the rest, 6 draws, and 12 losses. The two null scores are because no change point was supposed to be found in the corresponding time series.

The results obtained in this benchmark restate that our proposed method is promising, having a performance that competes with several state-of-the-art methods in the problem of novelty segmentation. It should be stressed that the proposed method applies to multidimensional time series, while two of the best-ranked methods in Figure 10 do not. In addition, the proposed method retrieves not solely segmentation points but also higher-level information as periodic changes and cross-segment similarity measures, which is an advantage over the *BOCPD*.

### 5.3. Time Complexity

In terms of computation time, the algorithm performs (1) a sliding window to extract features, which is *O(n)* complexity and (2) performs the dot product between matrices, which is traditionally *O($m^{2}n$)* (recall that *r* is the number of features and *n* is the size of the inputted signal). Finally, the correlation of a kernel on the SSM’s diagonal has a complexity of $O(nM^{2})$ (recall that M=2L+1, being the size of the sliding kernel).

The sliding window to extract features has an O(n) time complexity. The dot product between matrices has a conventional $O(m^{2}n)$ time complexity. Expressly, *m* and *n* represent the number of features and the inputted signal’s size, respectively, in our proposed method. The correlation computation of a kernel on the SSM’s diagonal has a complexity of $O(nM^{2})$, where the sliding kernel’s size *M* equals 2L+1.

### 5.4. Overall Discussion

Several parameters affect the detection results of desired patterns, especially the window size, the overlap percentage, and the kernel size, which influence visual outputs and the novelty function. These parameters can be explained with the analogy of a camera:

- The window size works like the *zoom function*, defining the scale of interest in the time series. Larger windows, corresponding to lower *zoom values*, allow the similarity calculation of longer *subsequences*, while smaller windows, serving like a *zoom-in* function, search for local details and unobtrusive changes.
- The overlap percentage, working as a down-sampler of the time series, is the *camera sensor*, which determines the image’s pixel resolution. A full resolution of the SSM is only achieved with total overlap, and the lower the overlap percentage, the less accurate are the highlighted changes.
- The sliding kernel’s size concerns the novelty function’s sharpness of the detected changes. The larger it is, the smoother the output function will be. Potentially, the kernel size can be scaled to the window size, even with a slight accuracy decrease.

With enough computational resources available, the overlap percentage can be maximized so that the SSM can mirror the full details. Admittedly, such an operation is not necessary for many real applications but reduces the variables to facilitate other parameters’ tuning experiments, which is one of our subsequent research topics. The computational resource, i.e., the memory bandwidth and the calculation time (see Section 5.3) is a limitation in this current stage since the SSM increases exponentially with the increase in the time series size. We ascertained that downsampling the time series with a lower overlap percentage is a valid option, advanced by a hierarchical search strategy for addressing the memory limitation, as exemplified in the walking-series instance in Section 5.1.1. Another potential efficiency-enhancing solution is only computing the SSM’s central diagonal with the kernel size corresponding to the interest areas of segmentation borders, which obtains efficiency gains in exchange for the sacrifice of periodicity and similarity measures between *subsequences*.

**Figure 11:**
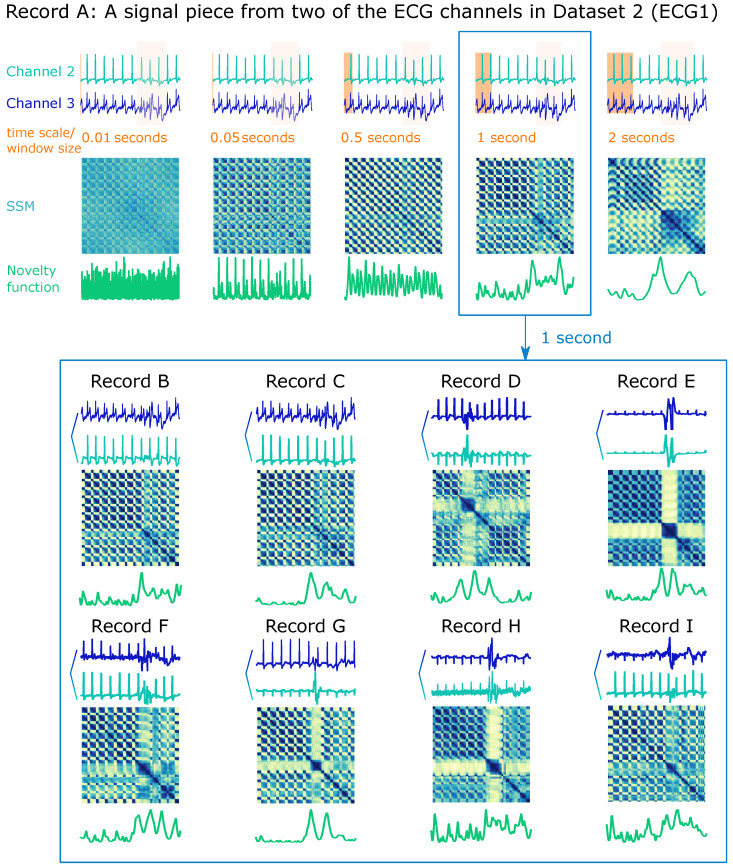
An illustrative example of window length intuition on records of Dataset 2 (ECG1, see Section 3.2). Top: different SSMs on the same ECG record A computed with sequentially larger window lengths from 0.01 to 2 s. The novelty functions are calculated with a kernel size equal to the window size and an overlap of 95%. Bottom: The 1-second window length is further applied as an example to indicate that parameters turned in the representation experiments can be generalized to all other records of the same dataset (B-I) to compute their corresponding SSM representations and novelty functions.

A reasonable intuition based on the understanding of signal characteristics should help configure the parameters mentioned above that are fundamental for computing the SSM and the novelty function, as Figure 11 demonstrates a starter example for segmentation purposes. The upper part of Figure 11 draws different SSMs on the same ECG record (A) from Dataset 2 (ECG1, see Section 3.2) computed with sequentially larger window lengths from 0.01 to 2 s. The appropriate window length depends on the purpose of the search:

- If a small window length, e.g., 0.05 s, is chosen, the novelty function will mostly detail changes within a heartbeat.
- If opting for a window length approximately equal to the ECG’s *PQRS* complex, each transition between complexes will be projected.
- If even larger windows are applied, e.g., 1 or 2 s, the jump artefact will be more significant on the SSM and be spotlighted on the novelty function. Hence, in such a case, the window length of 1 second should be appropriate for segmenting clean versus noisy ECG signals.

When using the same window length on all the other records of the same dataset, the SSM is expected to highlight the same regions of interest. Figure 11 (bottom) characterizes that parameters can be identical when working on the same data type and purpose, but the peak selection on the novelty function is not a matter of convention, which depends on the preset threshold.

The threshold used to determine which peaks are considered points of interest is not relevant to the SSM calculations but is closely related to event detection and automatic segmentation. If the data are a black box, the choice of threshold is a matter of observation and speculation. With informed knowledge of the data, the threshold can be predetermined and experimented with rules based on ranking the detected peaks from highest to lowest:

- Set the total number (or quantity range) of points of interest as the threshold. e.g., an ECG series with *x* heartbeats; an ACC series with *y* recorded gaits of walking activity.
- Count the total number of peaks and specify a percentage as the threshold. This work takes such an approach because of the diverse datasets and signals involved.
- Add sliding windows to the novelty function based on the known periodicity information of the time series, and define the number of points of interest expected to be present in each window as the threshold.

Typical signals in specialized fields should have approximately uniform methods and metrics for setting thresholds, and related follow-up studies are on the agenda.

## 6. Conclusions and Future Work

This article put forward a method based on the self-similarity matrix (SSM) for information retrieval of multimodal time series, with more interest in segmentation and further applications in automatic labelling. The proposed method uses a feature representation of the time series, arguing that a change in the signal can be detected by searching for differences in all the feature dimensions. The presented strategy requires three main parameters that can potentially be reduced to only the sliding window size, which is domain-agnostic and works with multidimensional time series, providing transparent visual intuition for the dynamics of the data. Furthermore, the SSM contains information for the posterior analysis of the *subsequences* segmented according to the detected events, thus enabling additional advanced applications, such as clustering/labelling based on the distance measures available on the SSM for each *subsequence* and summarizing the time series profiles of segmentability, periodicity and similarity. The SSM’s computational resource consumption can be reduced by setting variables based on available knowledge of the study objects and fields of interest.

Various application scenarios and types of signals involved in this work validated our proposed method’s high feasibility and usability by witnessing its capacity for novelty segmentation with remarkable performance that stands out among state-of-the-art methods. It is foreseeable that traditional video-based time-series segmentation tools could be upgraded to be more efficient and accessible with the aid of our proposed method.

The future work is widely branching out. A range of studies targeting the effective use of features, such as feature selection, feature stacking, feature space reduction, and high-level feature design, are crucial domain-related research topics. For example, when orienting the research field of HAR, the previous findings of [38,85,98] can be drawn upon in further experiments. Particular pattern changes in certain types of time series may be detected efficaciously by specific features or combinations thereof. Further studies also include, among others, similarity profiles-based automatic segmentation via the extensive use of our promising method, hierarchical segmentation for saving computational resources and better structural analysis, automatic clustering and information summarisation.

One of the most critical efforts should be put into investigating the association between parameters used for the detection of events, namely the window size, the kernel size and the overlap percentage, to reduce the parameter numbers, limit the ranges of attempts, or even provide reference parameter values for different domain-specific signals utilizing fixed-variable and greedy approaches. As we mentioned in Section 5.4, while the overlap percentage can be maximized to save one parameter in the experiments, there is potentially a relationship between the window size and the kernel size, which should be further explored and revealed with domain specificity.

Interfaceisation and softwareisation will enable the proposed method to be widely and practically accessible to help data users from all walks of life. For instance, using a scrollbar to expediently adjust various parameters for the intuitive observation of the SSM pattern transition and the event detection results will bring a qualitative leap forward in various research works, such as data mining, information retrieval, temporal structural analysis, and automatic segmentation and labelling. 

## Figures and Tables

**Table 1:** Results obtained by experiments with the novelty function-based, window-based, and binary segmentation approaches on different datasets, including true positives (TP), false positives (FP) and false negatives (FN), precision (P), recall (R) and F1-score (F1) values. The last row provides the macro averaged F1-scores (M.A. F1) of the four datasets.

	Novelty Function-Based						Window-Based						Binary					
Dataset	TP	FP	FN	P	R	F1	TP	FP	FN	P	R	F1	TP	FP	FN	P	R	F1
(1) HAR	166	16	13	0.91	0.93	0.92	169	9	10	0.95	0.94	0.95	125	58	54	0.68	0.70	0.69
(2) ECG1	18	1.0	0	0.95	1.00	0.97	13	5.0	5	0.72	0.72	0.72	16	2.0	2	0.89	0.89	0.89
(3) ECG2	155	9.0	13	0.95	0.92	0.93	139	13.0	29	0.91	0.83	0.87	122	58.0	46	0.68	0.73	0.70
(4) EMG	695	68	33	0.91	0.95	0.93	608	123	120	0.83	0.84	0.83	351	413	377	0.46	0.48	0.47
M.A. F1	-	-	-	-	-	0.94	-	-	-	-	-	0.84	-	-	-	-	-	0.69

**Table 2:** Points table recording segmentation match between the novelty-based (novelty), window-based (WL), and binary (BS) segmentation approaches on different datasets in the format of “Win/Draw/Lose” based on F1-scores. The last row reports the points for all datasets.

	Novelty Function-Based			Window-Based			Binary		
Dataset	Wins	Draws	Losses	Wins	Draws	Losses	Wins	Draws	Losses
(1) HAR1	0	0	2	2	0	0	0	0	2
(2) ECG1	2	7	0	0	4	5	0	6	3
(3) ECG2	10	1	1	0	0	12	1	1	10
(4) EMG	31	2	3	3	2	31	0	0	36
Overall	3	0	1	1	0	3	0	0	4

**Table 3:** Comparison of the F1-scores between our proposed method (*novelty*) and other algorithms’ benchmarks in Datasets 7 (ECG Pulsus Paradoxus, see Section 3.5). The calculation of all one-dimensional signals’ average performance and all signals’ average performance does not include columns with a grey background where no change point should be detected, or a signal error was present. Bold values represent the best F1-score for this specific dataset. T: timed out; F: failed compiling.

Dataset	novelty	amoc	binseg	bocpd	bocpdms	cpnp	ecp	kcpa	pelt	prophet	rbocpdms	rfpop	segneigh	wbs	zero
One-dimensional															
bank	0	**1.000**	**1.000**	**1.000**	0.500	0.054	0.200	0.333	0.400	**1.000**	T	0.015	**1.000**	0.043	**1.000**
bitcoin	0.694	0.507	0.690	0.733	0.533	0.611	0.625	0.665	**0.735**	0.446	T	0.284	**0.735**	0.690	0.450
brent_spot	**0.861**	0.465	0.670	0.609	0.239	0.607	0.636	0.553	0.586	0.249	T	0.521	0.586	0.564	0.315
businv	**0.927**	0.588	0.588	0.588	0.455	0.386	0.370	0.294	0.490	0.275	0.370	0.261	0.588	0.289	0.588
centralia	0.984	0.909	**1.000**	**1.000**	**1.000**	**1.000**	0.909	**1.000**	**1.000**	0.763	0.846	**1.000**	**1.000**	0.556	0.763
children_per_woman	**0.879**	0.678	0.663	0.712	0.405	0.344	0.551	0.525	0.637	0.310	0.504	0.246	0.637	0.500	0.507
co2_canada	0.851	0.544	0.856	**0.924**	0.479	0.642	0.875	0.867	0.670	0.482	0.542	0.569	0.872	0.681	0.361
construction	**0.933**	0.696	0.709	0.709	0.410	0.602	0.709	0.634	0.709	0.324	0.340	0.185	0.709	0.523	0.696
debt_ireland	0.974	0.760	**1.000**	**1.000**	0.892	0.958	0.980	**1.000**	**1.000**	0.469	0.748	0.824	**1.000**	0.538	0.469
gdp_argentina	**0.968**	0.889	0.947	0.947	0.583	0.818	0.889	0.800	0.947	0.615	0.452	0.615	0.947	0.421	0.824
gdp_croatia	**1.000**	**1.000**	0.824	**1.000**	0.583	**1.000**	0.824	0.583	0.824	0.824	0.824	0.400	0.824	0.167	0.824
gdp_iran	**0.921**	0.696	0.652	0.862	0.492	0.620	0.824	0.734	0.808	0.652	0.737	0.636	0.808	0.576	0.652
gdp_japan	**1.000**	**1.000**	0.889	**1.000**	0.615	0.667	**1.000**	0.500	0.889	0.889	0.889	0.222	0.889	0.222	0.889
global_co2	0.625	**0.929**	**0.929**	0.889	0.458	0.667	**0.929**	0.667	**0.929**	0.463	0.547	0.293	**0.929**	0.250	0.846
homeruns	**0.933**	0.812	0.829	0.829	0.650	0.650	0.829	0.829	0.812	0.723	0.397	0.661	0.812	0.664	0.659
iceland_tourism	0.652	0.947	0.947	0.947	0.486	0.391	**1.000**	0.486	0.643	0.220	0.667	0.200	0.947	0.200	0.947
jfk_passengers	**0.978**	0.776	0.776	0.776	0.650	0.602	0.651	0.437	0.776	0.354	T	0.491	0.776	0.437	0.723
lga_passengers	0.885	0.561	0.620	0.704	0.563	0.606	**0.892**	0.526	0.537	0.366	T	0.592	0.537	0.674	0.535
measles	0	**0.947**	**0.947**	**0.947**	0.486	0.118	0.080	0.281	0.153	0.391	F/T	0.030	**0.947**	0.041	**0.947**
nile	**1.000**	**1.000**	**1.000**	**1.000**	0.800	**1.000**	**1.000**	0.824	**1.000**	0.824	0.667	**1.000**	**1.000**	**1.000**	0.824
ozone	0.857	0.776	0.723	0.857	0.778	0.750	**1.000**	0.667	**1.000**	0.723	0.651	0.429	**1.000**	0.286	0.723
quality_control_1	**1.000**	**1.000**	**1.000**	**1.000**	0.667	0.667	**1.000**	0.667	**1.000**	0.500	0.286	0.667	**1.000**	0.667	0.667
quality_control_2	**1.000**	**1.000**	**1.000**	**1.000**	0.667	**1.000**	**1.000**	**1.000**	**1.000**	0.750	.429	**1.000**	**1.000**	**1.000**	0.750
quality_control_3	**1.000**	**1.000**	**1.000**	**1.000**	0.766	0.571	**1.000**	**1.000**	**1.000**	0.667	T	0.800	**1.000**	**1.000**	0.667
quality_control_4	**0.974**	0.810	0.873	0.787	0.561	0.658	0.726	0.658	0.780	0.780	T	0.241	0.780	0.608	0.780
quality_control_5	**0**	**1.000**	**1.000**	**1.000**	**0.500**	**1.000**	**1.000**	**1.000**	**1.000**	**1.000**	**0.500**	**1.000**	**1.000**	**1.000**	**1.000**
rail_lines	0.909	0.846	0.846	**0.966**	0.889	**0.966**	**0.966**	0.800	0.846	0.537	0.730	0.615	0.889	0.205	0.537
ratner_stock	0.933	0.776	0.824	0.868	0.559	0.396	0.776	0.754	0.824	0.280	T	0.203	0.824	0.378	0.571
robocalls	**0.979**	0.800	0.966	0.966	0.750	0.862	0.966	0.966	0.966	0.636	0.846	0.714	0.966	0.714	0.636
scanline_126007	0.887	0.710	0.920	**0.921**	0.829	0.906	0.870	0.838	0.889	0.644	T	0.649	0.889	0.818	0.644
scanline_42049	**0.977**	0.485	0.879	0.962	0.889	0.713	0.910	0.908	0.910	0.269	T	0.460	0.910	0.650	0.276
seatbelts	0.659	0.824	**0.838**	0.683	0.583	0.735	0.683	0.621	0.683	0.452	0.383	0.563	0.735	0.583	0.621
shanghai_license	**0.979**	0.966	0.868	0.868	0.605	0.600	0.868	0.465	0.868	0.532	0.389	0.357	0.868	0.385	0.636
uk_coal_employment	F	F	F	F	0.617	F	0.513	0.513	F	**0.639**	F	F	F	F	0.513
unemployment_nl	0.820	0.742	**0.889**	0.876	0.592	0.747	0.755	0.744	0.788	0.566	F/T	0.628	0.788	0.801	0.566
us_population	0.636	**1.000**	0.889	**1.000**	0.615	0.232	0.471	0.276	0.500	0.159	T	0.889	0.889	0.113	0.889
usd_isk	**0.914**	0.785	0.704	0.785	0.678	0.674	0.785	0.601	0.657	0.489	0.510	0.462	0.678	0.636	0.489
well_log	0.814	0.336	0.914	0.832	0.743	0.822	**0.928**	0.776	0.873	0.149	T	0.923	0.873	0.832	0.237
**Average F1-measure (1D)**	**0.845**	0.739	0.798	0.822	0.596	0.651	0.784	0.657	0.766	0.482	0.354	0.517	0.797	0.517	0.599
Multidimensional															
apple	**0.949**			0.916	0.445		0.745	0.634			F/T				0.594
bee_waggle_6	0.657			**0.929**	0.481		0.233	0.634			0.245				**0.929**
occupancy	**0.953**			0.919	0.735		0.932	0.812			F/T				0.341
run_log	0.994			**1.000**	0.469		0.990	0.909			0.380				0.446
**Average F1-measure (ALL)**	**0.871**	n.a.	n.a.	0.855	0.604	n.a.	0.797	0.683	n.a.	n.a.	0.343	n.a.	n.a.	n.a.	0.61
**WINS (ALL)**	16	0	2	2	0	0	3	0	0	0	0	0	0	0	0
**DRAWS (ALL)**	6	9	8	11	1	6	8	4	9	1	0	3	8	2	0
**LOSES (ALL)**	12	25	24	21	33	28	23	30	25	33	34	31	26	32	34

## Data Availability

All datasets used in this work are publicly available. Please refer to the link to access each of the used datasets (all accessed on 30 September 2022): HAR—https://archive.ics.uci.edu/ml/datasets/human+activity+recognition+using+smartphones; ECG1—https://physionet.org/content/macecgdb/1.0.0/; ECG2—https://physionet.org/content/nstdb/1.0.0/; EMG—https://archive.ics.uci.edu/ml/datasets/EMG+data+for+gestures; ATCPD—https://github.com/alan-turing-institute/TCPDBench; ABP—https://physionet.org/content/prcp/1.0.0/; ECGPulsus—signal from [60].

## Abbreviations

The following abbreviations are used in this manuscript:

ABP	Arterial Blood Pressure
ACC	Accelerometer
BINSEG	Binary Segmentation
BOCPD	Bayesian Online Change Point Detection
BS	Binary Segmentation
BVP	Blood Volume Pressure
ECG	Electrocardiogram
ED	Euclidean Distance
EEG	Electroencephalogram
EMG	Electromyogram
EOG	Electrocularogram
FLOSS	Fast Low-cost Online Semantic Segmentation
FN	False Negative
FP	False Positive
GYR	Gyroscope
HAR	Human Activity Recognition
HMM	Hidden Markov Model
IMU	Inertial Motion Unit
LSTM	Long Short-Term Memory
NN	Neural Networks
RESP	Respiratory Inductance Pletismography
SegNeigh	Segment Neighbourhood
SNR	Signal-to-Noise-Ratio
SSM	Self-Similarity Matrix
TN	True Negative
TP	True Positive
TSFEL	Time Series Feature Extraction Library
TSSEARCH	Time Series Subsequence Search Library
WS	Window-Based Segmentation

## Appendix A. Feature List, Parameter Configurations, and Statistical Results of Segmentation Experiments

**Table A1:** Features applied in this work for creating the SSMs. The Time Series Feature Extraction Library (TSFEL) is utilized for feature extraction.

Temporal Domain	Statistical Domain	Frequency Domain
Absolute energy	Interquartile Range	Entropy
Area under the curve	Kurtosis	Fundamental frequency
Centroid	Maximum	Max frequency
Cumulative centroid	Mean	Roll off
Distance	Mean absolute deviation	Roll on
Maximum peak	Median	Spectral distance
Mean absolute difference	Minimum	Spectral kurtosis
Mean difference	Root mean square	Spectral skewness
Median absolute difference	Skewness	Spectral spread
Total energy	Standard deviation	
	Variance	

**Table A2:** Parameter configuration and experimental results of each signal in Dataset 1 (HAR, see Section 3.1). $W_{size}$: window size; K%: kernel size in percentage of the window size; O%: overlap percentage of the window size. T%: amplitude threshold for event detection; WS: window-based segmentation; BS: binary segmentation. The tolerance used to calculate the F1-score (F1) equals the window size for novelty function computation.

	Novelty Function-Based					WS		BS
Signal	$W_{\mathrm{size}}$	K%	O%	T%	F1	$W_{\mathrm{size}}$	F1	F1
1	1500	1.5	0.90	0.01	0.91	1500	0.95	0.68
2	1500	1.5	0.90	0.01	0.94	1500	0.95	0.72

**Table A3:** Parameter configuration and experimental results of each signal in Dataset 2 (ECG1, see Section 3.2). $W_{size}$: window size; K%: kernel size in percentage of the window size; O%: overlap percentage of the window size. T%: amplitude threshold for event detection; WS: window-based segmentation; BS: binary segmentation. The tolerance used to calculate the F1-score (F1) equals the window size for novelty function computation.

	Novelty Function-Based					WS		BS
Signal	$W_{\mathrm{size}}$	K%	O%	T%	F1	$W_{\mathrm{size}}$	F1	F1
0	400	3.74	0.55	0.95	1.0	500	0.5	0.5
1	500	2.59	0.95	0.95	1.0	400	1.0	1.0
2	150	4.00	0.82	0.95	0.8	200	0.5	0.5
3	150	2.66	0.32	0.95	1.0	300	1.0	1.0
4	300	2.66	0.32	0.95	1.0	300	1.0	1.0
5	300	2.66	0.59	0.95	1.0	150	0.5	1.0
6	150	2.66	0.64	0.95	1.0	400	0.5	1.0
7	150	2.66	0.28	0.95	1.0	500	1.0	1.0
8	150	2.66	0.55	0.95	1.0	200	0.5	1.0

**Table A4:** Parameter configuration and experimental results of each signal in Dataset 3 (ECG2, see Section 3.3). $W_{size}$: window size; K%: kernel size in percentage of the window size; O%: overlap percentage of the window size. T%: amplitude threshold for event detection; WS: window-based segmentation; BS: binary segmentation. The tolerance used to calculate the F1-score (F1) equals the window size for novelty function computation.

	Novelty Function-Based					WS		BS
Signal	$W_{\mathrm{size}}$	K%	O%	T%	F1	$W_{\mathrm{size}}$	F1	F1
1	12,500	0.63	0.14	0.95	1.00	50,000	0.96	0.97
2	12,500	1.25	0.19	0.95	0.97	50,000	0.96	0.97
3	25,000	2.50	0.10	0.95	0.92	50,000	0.92	0.83
4	50,000	0.62	0.28	0.95	0.93	50,000	0.85	0.48
5	50,000	0.62	0.14	0.95	0.67	50,000	0.64	0.34
6	12,500	0.63	0.14	0.95	1.00	50,000	0.96	0.90
7	12,500	1.25	0.19	0.95	1.00	50,000	0.96	0.90
8	12,500	1.25	0.23	0.95	1.00	25,000	0.93	0.90
9	12,500	1.25	0.28	0.95	1.00	25,000	0.86	0.55
10	12,500	1.25	0.32	0.95	0.96	25,000	0.71	0.34
11	25,000	2.50	0.41	0.95	0.75	50,000	0.67	0.34
12	12,500	1.25	0.10	0.95	1.00	50,000	0.96	0.90

**Table A5:** Parameter configuration and experimental results of each signal in Dataset 4 (EMG, see Section 3.4). $W_{size}$: window size; K%: kernel size in percentage of the window size; O%: overlap percentage of the window size. T%: amplitude threshold for event detection; WS: window-based segmentation; BS: binary segmentation. The tolerance used to calculate the F1-score (F1) equals the window size for novelty function computation.

	Novelty Function-Based					WS		BS
Signal	$W_{\mathrm{size}}$	K(%)	O(%)	T(%)	F1	$W_{\mathrm{size}}$	F1	F1
1	2000	0.62	0.95	0.19	0.90	2000	0.72	0.29
2	2000	1.25	0.95	0.28	0.86	1500	0.75	0.39
3	1500	1.27	0.95	0.10	1.00	2000	0.95	0.78
4	1500	0.63	0.95	0.14	1.00	2000	0.89	0.49
5	2000	1.25	0.95	0.28	0.92	1500	0.85	0.20
6	1500	0.63	0.95	0.28	0.88	1500	0.74	0.29
7	2000	1.25	0.95	0.19	0.95	2000	0.95	0.49
8	1500	1.27	0.95	0.10	0.93	2000	0.85	0.63
9	2000	0.62	0.95	0.28	1.00	2000	0.95	0.44
10	2000	1.25	0.95	0.28	0.97	1500	0.90	0.54
11	1000	1.25	0.95	0.10	1.00	2000	0.96	0.82
12	2000	1.25	0.95	0.14	0.83	2000	0.85	0.34
13	1000	0.62	0.95	0.37	1.00	2000	0.75	0.78
14	1000	1.25	0.95	0.32	0.95	1500	0.65	0.29
15	2000	0.62	0.95	0.14	0.93	1500	0.85	0.54
16	2000	0.62	0.95	0.28	0.90	2000	0.81	0.39
17	1500	2.53	0.95	0.10	0.98	2000	0.83	0.29
18	2000	0.62	0.95	0.50	0.97	1500	0.90	0.49
19	2000	0.62	0.95	0.23	0.93	1500	0.75	0.44
20	1500	0.63	0.95	0.10	0.95	2000	0.95	0.59
21	2000	1.25	0.95	0.19	0.95	2000	0.95	0.44
22	2000	1.25	0.95	0.19	0.84	2000	0.90	0.29
23	1500	1.27	0.95	0.28	0.90	1500	0.85	0.54
24	2000	0.62	0.95	0.19	0.86	2000	0.76	0.39
25	1500	1.27	0.95	0.37	0.95	2000	0.85	0.68
26	1500	0.63	0.95	0.19	0.98	2000	0.90	0.54
27	1500	1.27	0.95	0.19	0.83	1500	0.75	0.59
28	1500	1.27	0.95	0.37	0.90	1500	0.80	0.29
29	1500	1.27	0.95	0.23	1.00	2000	0.76	0.34
30	2000	2.50	0.95	0.10	0.92	2000	0.83	0.20
31	1000	1.25	0.95	0.19	1.00	1500	0.90	0.73
32	1500	1.27	0.95	0.41	0.89	2000	0.77	0.29
33	2000	0.62	0.95	0.23	0.83	1500	0.80	0.63
34	2000	0.62	0.95	0.28	0.93	1000	0.70	0.34
35	1500	0.63	0.95	0.14	0.95	2000	0.84	0.83
36	1000	2.50	0.95	0.14	1.00	1500	0.80	0.29

**Table A6:** Parameter configuration and novelty function-based experimental results of each signal in Dataset 5 (CPDBenchmark, see Section 3.5). $W_{size}$: window size; K%: kernel size in percentage of the window size; T%: amplitude threshold for event detection; The tolerance corresponded to five samples, specified by the benchmark specifications [8].

Signal	F1	$W_{\mathrm{size}}$	K%	T%
apple	0.95	10	50	0.65
bank	0.67	100	100	0.90
bee_waggle_6	0.66	100	250	0.80
bitcoin	0.69	10	65	0.15
brent_spot	0.86	10	30	0.75
businv	0.93	20	15	0.70
centralia	0.98	6	2	0.70
children_per_woman	0.88	10	10	0.70
co2_canada	0.85	10	20	0.35
construction	0.93	20	50	0.70
debt_ireland	0.97	6	2	0.70
gdp_argentina	0.97	20	10	0.70
gdp_croatia	1.00	20	10	0.70
gdp_iran	0.92	10	10	0.70
gdp_japan	1.00	10	10	0.70
global_co2	0.62	100	50	0.70
homeruns	0.93	15	25	0.50
iceland_tourism	0.65	150	150	0.99
jfk_passengers	0.98	20	30	0.80
lga_passengers	0.89	20	30	0.80
measles	0.17	20	30	0.80
nile	1.00	20	30	0.80
occupancy	0.95	10	20	0.40
ozone	0.86	6	4	0.60
quality_control_1	1.00	6	20	0.60
quality_control_2	1.00	6	20	0.60
quality_control_3	1.00	20	30	0.80
quality_control_4	0.97	10	50	0.75
rail_lines	0.91	6	2	0.75
ratner_stock	0.93	6	50	0.75
robocalls	0.98	6	10	0.50
scanline_126007	0.89	6	10	0.30
scanline_42049	0.98	6	20	0.50
seatbelts	0.66	6	20	0.30
shanghai_license	0.98	20	10	0.80
unemployment_nl	0.82	4	6	0.27
usd_isk	0.91	6	25	0.30
us_population	0.93	4	6	0.27
well_log	0.81	10	15	0.40
